# Heart-breaking tumours: a case series of malignant pericardial effusion

**DOI:** 10.1093/ehjcr/ytaf009

**Published:** 2025-01-18

**Authors:** Syarifah Syafiqah Wan Muhamad Hatta, Abdur Rahman Mirza, Nadia Sunni, Ahmed Bashir

**Affiliations:** Department of Cardiology, Walsall Healthcare NHS Trust, Walsall, UK; University of Birmingham Medical School Vincent Drive, Edgbaston, Birmingham B15 2TT, UK; Department of Cardiology, Walsall Healthcare NHS Trust, Walsall, UK; Department of Cardiology, Walsall Healthcare NHS Trust, Walsall, UK

**Keywords:** Pericardial tumour, Pericardial mesothelioma, Pericardial sarcoma, Pericarditis, Pericardial biopsy, Cardiac magnetic resonance imaging, Case series

## Abstract

**Background:**

Malignant pericardial effusions are often linked to metastases from solid tumours, such as those in the lung or breast, or haematological diseases. Primary cardiac tumours are rare, occurring in only 0.02% of cases, with pericardial tumours comprising 6.7%–12.8% of all primary cardiac tumours.

**Case summary:**

In Case 1, a 49-year-old Black African male presented with chest pain and breathlessness after a COVID-19 vaccine. Initially treated for pericarditis, he returned with worsening symptoms. Echocardiography revealed pericardial effusion and cardiac tamponade. Imaging confirmed a right atrial mass diagnosed as malignant biphasic mesothelioma. He died 4 months after diagnosis. In Case 2, a 43-year-old Caucasian male developed breathlessness and fever post-COVID-19 vaccine. Imaging identified a large posterior pericardial mass, later diagnosed as synovial sarcoma. Chemotherapy yielded minor tumour reduction, but he succumbed to his illness, spending his final days in a hospice.

**Discussion:**

Initial clinical signs are critical in determining the origin of pericardial effusion. Malignancy should be suspected in cases with cardiac tamponade, unexplained haemorrhagic pericardial fluid, or recurrent symptoms. Negative cytology warrants further investigation with advanced imaging or biopsy to improve diagnostic sensitivity. Diagnosing rare tumours involves multiple imaging modalities, fluid analysis, biopsies, and an interdisciplinary approach, with pathological analysis being the gold standard. Treatment remains challenging due to the rapid progression of these tumours, with surgery often not feasible. A multi-pronged diagnostic approach is crucial, and clinicians must maintain suspicion for malignancy in persistent pericardial effusion cases, even in the context of other potential confounding factors.

**Learning points**

- Highlight the significance of maintaining a high suspicion of malignancy when encountering acute pericardial disease with cardiac tamponade, unexplained haemorrhagic pericardial fluid, and a persistent or recurrent course.
- Do not rely solely on negative pericardial cytology results; if necessary, pursue additional investigations, including a pericardial biopsy, to enhance the overall diagnostic sensitivity for pericardial malignancy, especially in rare tumours.
- Address the diagnostic challenges in these case series, such as misleading clinical presentations of pericardial disease like pericarditis, and the difficulties in obtaining a diagnostic pericardial sample in cases of rare tumours.

## Introduction

Pericardial effusion is the accumulation of excess fluid between the visceral and parietal pericardium, caused by idiopathic, viral, bacterial, autoimmune, or post-myocardial infarction conditions.^1^ Malignant pericardial effusions occur in 13%–23% of cases, often due to metastatic lung or breast cancer.^2^ Primary pericardial tumours, such as mesotheliomas and sarcomas, are rare.^3,4^ This case series highlights the diagnostic and therapeutic challenges of primary pericardial tumours and underscores key clinical lessons.

## Summary figure

**Figure:**
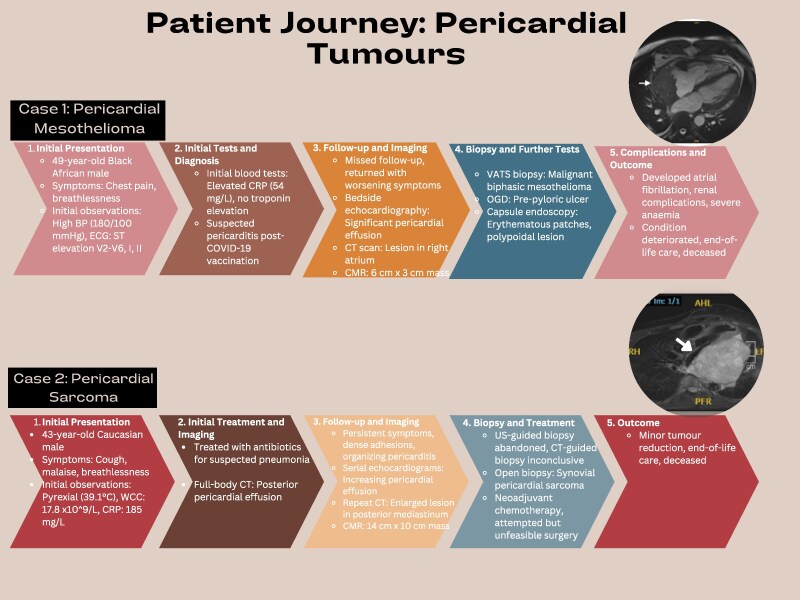


## Case 1

**Figure 1:**
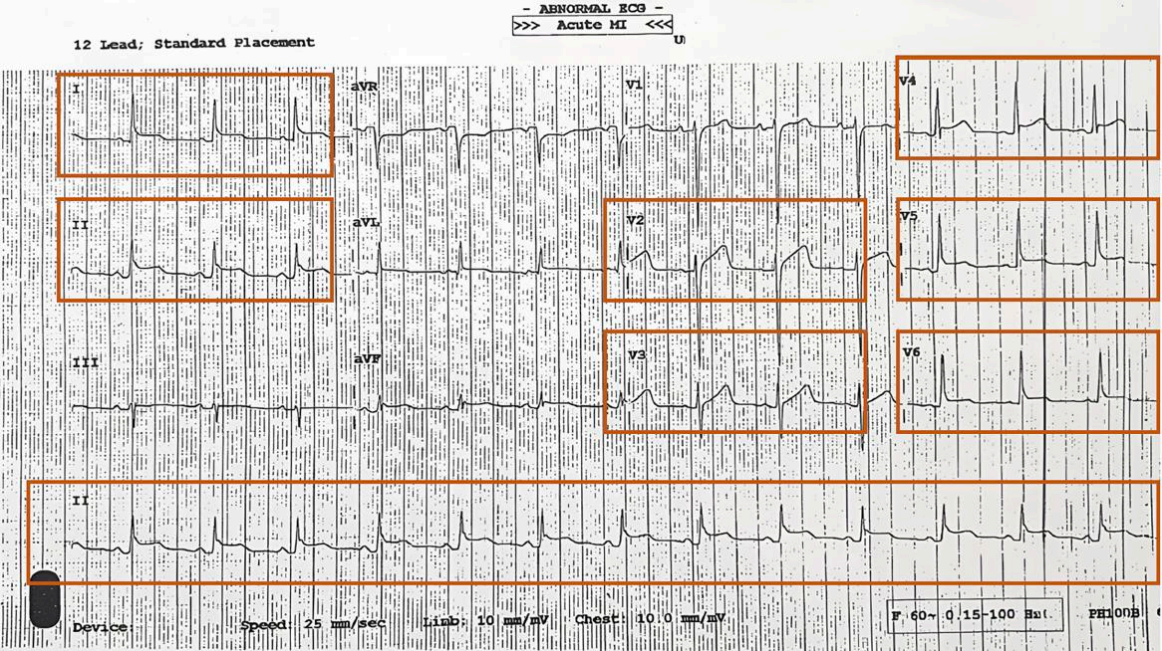
Admission ECG showing near-global ST elevation in Leads V2–V6, I, and II.

A 49-year-old Black African male presented with chest pain and breathlessness 4 weeks after his first Pfizer–BioNTech COVID-19 vaccine. Initial observations were normal except for a slightly high blood pressure (180/100 mmHg). ECG showed PR depression in Leads I, II, V4–V6 and near-global concave ST elevation in Leads V2–V6, I, and II (*Figure 1*).

Initial blood tests showed elevated c-reactive protein (CRP) (54 mg/L) (*Table 1*) but no serial elevation in serum troponin levels, suggesting suspected pericarditis secondary to the COVID-19 vaccination. Treatment focussed on managing as pericarditis.

**Table 1:** Initial blood test results on admission

Blood test	Admission	Range
Hb	99 g/L	120–180 g/L
Platelet	159 (10^9^/L)	150–4400 (10^9^/L)
White cell count	3.9(10^9^/L)	3.6–11 (10^9^/L)
Urea	6.5 mmol/L	2.5–7.8 mmol/L
Creatinine	99 μmol/L	59–1044 μmol/L
CRP	54 mg/L	>5 mg/L
First troponin	20 ng/L	<14 ng/L
Second troponin (3H)	20 ng/L	<14 ng/L
Brain natriuretic peptide	259 pg/mL	<400 pg/mL

The patient missed his follow-up echocardiogram and returned 4 weeks later with worsening breathlessness. Bedside echocardiography revealed a significant pericardial effusion and right ventricular collapse during diastole, indicative of cardiac tamponade requiring emergency drainage. The pericardial fluid was haemorrhagic but cytology was negative for malignancy. The patient worked in a cardboard factory with no asbestos exposure and had diabetes Type II and hypertension.

**Figure 2:**
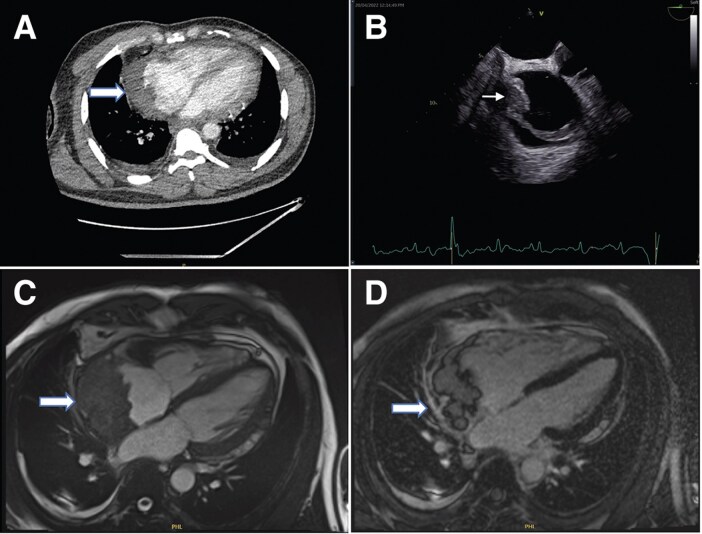
(*A*) Contrast computed tomography thorax (using Omnipaque 350 contrast agent) showing a filling defect in the right atrium’s lateral wall. (*B*) This was also seen in the transoesophageal echo as seen in the image on the right. (*C*) Four-chamber balanced SFPP Cine sequence revealed a large mass (6 × 3 cm) with irregular margins which appears to originate from the pericardium and compressing the right atrium. (*D*) Four-chamber delayed gadolinium enhancement sequence showed heterogeneous enhancement of the mass and no myocardial late gadolinium enhancement.

Computed tomography (CT) scan of the thorax, abdomen, and pelvis revealed a suspicious lesion in the right atrium’s lateral wall (*Figure 2A*), also confirmed by transoesophageal echocardiography (*Figure 2B* and Supplementary material, *Video 1*). An 18-fluorodeoxyglucose (FDG) Positron Emission Tomography CT scan showed intense FDG uptake in the right atrial mass without metastasis. Due to the small mass size, an intracardiac percutaneous biopsy was deemed low yield, so surgical excision biopsy was planned.

The patient missed interval follow-up reviews but returned months later. A repeat cardiac magnetic resonance (CMR) showed an increase in the mass size to 6 cm × 3 cm, with hyperintense signal intensity on T_2_, isointense on T_1_, and some enhancement on first-pass perfusion and late gadolinium images (*Figure 2C, D*). Imaging suggested a malignant primary cardiac or pericardial tumour.

**Figure 3:**
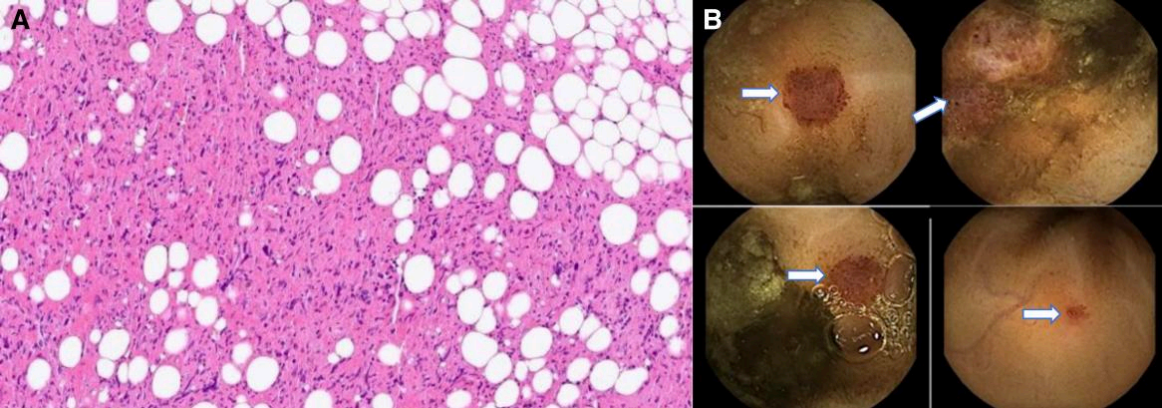
(*A*) Histopathology showing epithelioid and spindled cell proliferation. (*B*) Capsule endoscopy from the small bowel revealed multiple small erythematous patch-like lesions and a large erythematous polypoidal lesion.

For operability concerns due to tumour size and extent, a right video-assisted thoracic surgery (VATS) biopsy was performed. Histology indicated a malignant biphasic mesothelioma, with both epithelioid and spindled cells, mostly expressing mesothelial markers. The differential diagnosis included undifferentiated sarcoma with reactive mesothelial proliferation (*Figure 3A*).

Subsequently, the patient also underwent an oesophago-gastro-duodenoscopy (OGD) for significant recurring iron deficiency anaemia. The OGD showed a small pre-pyloric ulcer. Colonoscopy did not reveal any abnormality.

Due to the inconclusive findings, he underwent a capsule endoscopy, and the findings were intriguing. There were multiple small erythematous patches and a large polypoidal erythematous lesions with a high vascular content which did not appear to be angiodysplastic in nature (*Figure 3B*). Probable differential diagnoses included metastasis to the bowel, carcinoid, or Kaposi sarcoma. HIV test was negative and further pericardial biopsy tested for HHV-8 was negative making Kaposi unlikely.

Despite these intriguing findings, it remains uncertain whether there was a paraneoplastic manifestation or provided any significance to his overall disease progression.

The patient developed atrial fibrillation due to the likely compressive effects of the tumour, as well as renal complications, and severe anaemia. He died 4 months after diagnosis.

## Case 2

A 43-year-old Caucasian male with asthma and recent COVID-19 vaccination presented with a 3-week history of cough and breathlessness. On admission, he was pyrexial (39.1°C) with raised infection markers (white cell count of 17.8 ×10^9^/L, CRP of 185 mg/L). His ECG showed sinus tachycardia, and his chest radiograph indicated an enlarged cardiac silhouette.

**Figure 4:**
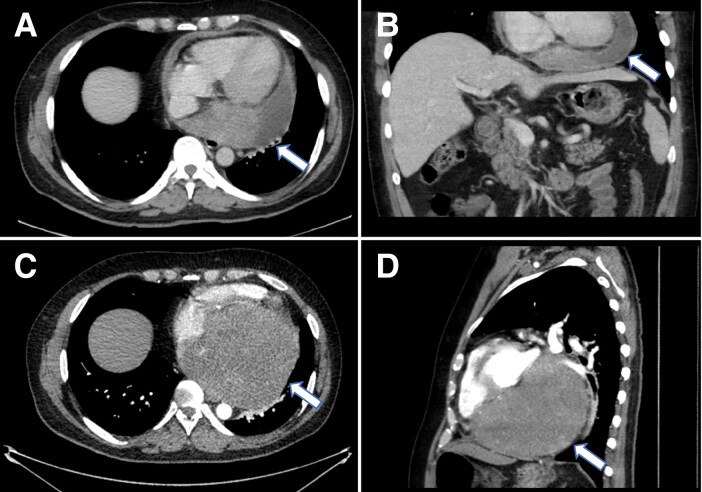
(*A*, *B*) Contrast computed tomography scan (using Omnipaque 350 contrast agent) showing a moderate-sized pericardial effusion predominantly accumulated in the posterior pericardial cavity, with a maximum depth of 4.5 cm. (*C*, *D*) Repeat contrast computed tomography scan (using Omnipaque 350 contrast agent) showed a significantly increased lesion in the pericardium compressing the left atrium and ventricle.

Initially treated for community-acquired pneumonia, his symptoms persisted despite antibiotics. Blood cultures were negative, and a full-body CT confirmed a large pericardial effusion in the posterior pericardial space (*Figure 4A, B*).

Echocardiography showed a large pericardial effusion of 4 cm posteriorly situated to the left side of the heart. Treatment with colchicine and antibiotics were ineffective. A VATS pericardial window was attempted but unsuccessful due to dense adhesions.

Serial echocardiograms over 8 months revealed increasing effusion size, leading to atrial compression and mitral regurgitation. A repeat contrast CT at 10 months showed a heterogeneously enhancing posterior mediastinal mass compressing the left atrium and ventricle (*Figure 4C, D*).

**Figure 5:**
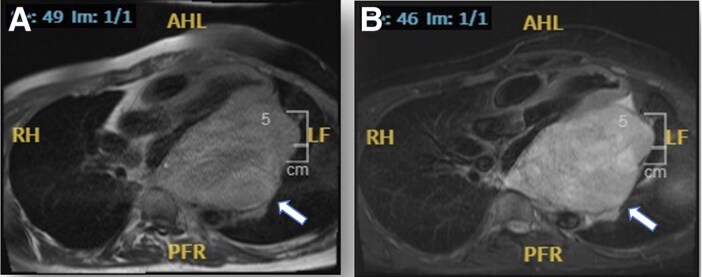
Cardiac magnetic resonance scan, three-chamber view showing 14 × 10 cm oval mass in pericardium towards the inferior surface of heart, compressing left atrium and inferior vena cava entrance to right atrium revealed heterogeneous increased signal intensity on T_1_ weighted turbo spin echo with fat suppression (*A*) and heterogeneous increased signal intensity on T_2_ weighted turbo spin echo (*B*).

CMR revealed a 14 cm × 10 cm mass in the pericardium towards the inferior surface of the heart. It has an increased signal intensity on T_1_ with fat suppression, and increased signal intensity on T_2_, with some early and late gadolinium enhancement. Tissue characterization was suggestive of a sarcoma (*Figure 5A, B*). An ultrasound guided biopsy was attempted and abandoned due to the location of the mass hence a CT-guided biopsy was performed but yielded inconclusive results.

The patient was referred for neoadjuvant chemotherapy followed by an attempted tumour resection via left postero-lateral thoracotomy. However, the highly vascular, myocardium-invading tumour made surgical excision unfeasible. An open biopsy confirmed synovial pericardial sarcoma. He received six cycles of chemotherapy (Doxorubicin and Ifosfamide), which resulted in some improvement and minor tumour reduction on follow-up CT.

Despite treatment, the patient succumbed to his illness, spending his final days in a hospice.

## Discussion

Initial clinical signs and symptoms are crucial in determining the origin of pericardial effusion. Cardiac tamponade without inflammatory signs triples the likelihood of an underlying malignancy.^5^ Similarly, patients with a history of malignancy are 20 times more likely to have an underlying malignant cause whilst those who have recurrent pericarditis or non-responsiveness to non-steroidal anti-inflammatory drugs are approximately 10 times more likely to have a neoplastic origin.^3^

Although asbestos exposure is a key risk factor in other types of mesotheliomas, there is currently no strong evidence to suggest an aetiology link for pericardial mesothelioma.^6^ Synovial sarcoma, however, is linked to the t(X;18)(p11;q11) genetic translocation, primarily affecting para-articular tissues in young adults but it is very rare for it to originate in the pericardium.^7,8^

Due to the rarity of primary pericardial malignancies, survival data is limited to case series and retrospective studies. A 2009 study reported a median survival of 6 months for pericardial mesothelioma, which aligns with our case.^6^ Another study reported a median survival of 27 months for pericardial synovial malignancies, consistent with our patient’s 24-month survival post-presentation.^9^

In case one, despite negative cytology, malignancy was suspected due to cardiac tamponade. A full-body scan revealed an ill-defined mass, and a pericardial biopsy confirmed pericardial mesothelioma. This underscores the need for further investigations and pericardial biopsy when cytology is negative, as it enhances diagnostic sensitivity for rare tumours.

Cytology alone carries a false negative rates of 4%–14.7%.^2,10^ Combining cytology with biopsy increases diagnostic sensitivity for malignancy by ∼8%.^11^ At least 60 mL of pericardial fluid is required for a cytologic diagnosis of malignant pericardial effusion, achieving a sensitivity of 91.7%–92.1%.^11^

Managing rare tumours requires multimodal imaging, fluid analysis, biopsies, and pathology, with an interdisciplinary approach. The cases demonstrate the effectiveness of these methods in distinguishing between benign and malignant tumours, with pathological analysis being the gold standard.

Treatment studies for these rare tumours are limited. Complete surgical resection is preferred, but the rapid, infiltrative nature of these tumours often precludes surgical intervention. For synovial sarcoma, combining ifosfamide and doxorubicin shows a 58.6% response rate compared to a single agent.^12^ Although our patient-reported symptom improvement, tumour shrinkage was minimal, making further surgical resection unfeasible.

Additionally, following the rise in the association between the COVID vaccine and myopericarditis, particularly in males aged 12–39,^13^ it is easy for clinicians to develop a diagnostic bias. This emphasizes the importance of revisiting the diagnosis if symptoms persist or present in a more acute manner.

## Supplementary Material

ytaf009_Supplementary_Data

## Lead author biography

Dr Syafiqah Hatta is in her final year of training in Internal Medicine at West Midlands, UK

## Supplementary material

**Consent:** Written informed consent was obtained from the patient for publication of this case report, including accompanying images in accordance with COPE guidelines.

**Funding:** The authors have no source of funding to declare for this manuscript.

**Data availability:** The data underlying this article are available in the article and its online Supplementary material.

## Publication

This manuscript has been published as a presentation^1^ and an abstract^2^ at two conferences as listed below:

1. Heart-breaking tumours: A case series of malignant pericardial effusions, was submitted to the ESC Congress in Amsterdam 2023. https://esc365.escardio.org/presentation/270198.
2. Hatta SSBWM, Mirza AR, Sunni N, Bashir A. A case series of malignant pericardial effusion. Clin Med (Lond). 2023 Nov;23(Suppl 6):18–19. doi: 10.7861/clinmed.23-6-s18. PMID: 38182251; PMCID: PMC11046613.
